# Result Assessment Tool (RAT): empowering search engine data analysis

**DOI:** 10.7717/peerj-cs.2962

**Published:** 2025-07-08

**Authors:** Sebastian Sünkler, Dirk Lewandowski, Sebastian Schultheiß, Nurce Yagci

**Affiliations:** 1 Department of Information, Media and Communication, Hamburg University of Applied Sciences, Hamburg, Germany; 2 Department of Computer Science and Applied Cognitive Science, University of Duisburg-Essen, Duisburg, Germany

**Keywords:** Search engine evaluation, Web scraping, Retrieval tests, Retrieval effectiveness studies, Search engines, Search engine optimization, Information science, Health information, Open source, Python

## Abstract

The Result Assessment Tool (RAT) is a Python-based software toolkit that enables researchers to analyze results from commercial search engines, social media platforms, and library search systems. RAT provides an integrated environment for designing studies, collecting results, and performing automated analysis. The software consists of two main modules: RAT Frontend and RAT Backend. RAT Frontend uses Flask to provide a researcher view for designing studies and an evaluation view for collecting ratings from study participants. RAT Backend includes modules for collecting search results, extracting source code, and adding classifiers for automated analysis. The system has been used in various studies, including search engine effectiveness studies, interactive information retrieval studies, and classification studies.

## Introduction

The aim of developing the Result Assessment Tool (RAT) software is to allow researchers to access and analyze results from different search systems. Collecting and analyzing such data is essential to researchers from various fields, including information science, health information, computer science, computational social sciences, and more.

For researchers to understand the modern information environment, access to data from platforms such as Facebook, Google, and X (Twitter) is paramount. While some of these platforms allow (paid) access to at least some of their data, search engine data is not available satisfactorily. Microsoft Bing is the only major search engine that provides interested parties with an application programming interface (https://www.microsoft.com/en-us/bing/apis/bing-web-search-api). However, as Google dominates the search engine market with 91 percent of global searches (StatCounter, 2024), researchers are interested in seeing which results users face and need access to Google data. Furthermore, many other search systems apart from web search engines do not provide access to their data but may interest researchers.

In the following, we will speak of *search engines* when a search system collects data from the web (for definitions, see Lewandowski, 2023). The more general term *search system* refers to any information retrieval system that allows a user to search a data collection through a web interface. It can refer to any website that incorporates a search functionality. Some examples of search systems include library search systems (https://catalog.loc.gov/vwebv/searchBrowse), product search (https://www.amazon.com/), social media networks (Twitter), and local search functions on any website.

Researchers may have different interests when it comes to studying search systems. On the one hand, they might focus on the results generated by a specific search system. In addition to general-purpose web search engines, these can include, among others, library search systems, news search engines, or video search engines. Here, researchers may be interested in the results a particular search system shows its users. On the other hand, researchers may want to compare different systems. For instance, researchers can compare two or more web search engines, vertical search engines (such as different news search engines), or web search engines with more specialized systems. These cases frequently include evaluating the retrieval effectiveness of various search engines and search systems and are common scenarios in the field of information science (*e.g.*, Lewandowski, 2015; Shafi & Ali, 2019; Gul, Ali & Hussain, 2020; Zeynali Tazehkandi & Nowkarizi, 2021; Maillé et al., 2022). Researchers would benefit from an integrated system for the cases mentioned, allowing them to design studies, collect results from different search systems, synthesize findings, and conduct analyses. We designed RAT to meet these needs.

However, RAT is not limited to evaluating search systems; it could also support research in various fields. For instance, in the health sector, RAT could assist in quality evaluations (*e.g.*, Janssen et al., 2018) and content analyses (*e.g.*, Rachul et al., 2020) of health-related search results. In media and communication studies, RAT could help classify content types and assess ideological bias (*e.g.*, Ballatore, 2015). The Results section provides a detailed description of how RAT could have been applied in these studies.

A significant methodological challenge in search engine research involves detecting and addressing biases in search results. Traditional manual collection methods often create three key problems: (1) temporal inconsistency, as results change over time; (2) sampling limitations that prevent statistical power; and (3) potential researcher bias in collection and classification. RAT directly addresses these methodological challenges in several ways. It enables researchers to systematically collect large datasets from multiple search engines simultaneously, capturing results precisely as they appear at a specific moment, and its standardized assessment interface ensures all evaluators apply consistent criteria when classifying content. RAT also offers manual and automated analysis tools that detect patterns across significant result sets that would be impossible to identify manually. These capabilities have been demonstrated in studies like Yagci et al. (2022) and Norocel & Lewandowski (2023), which used RAT to analyze source diversity and the presence of extreme content in mainstream search results—analyses that would be impractical with traditional methods. The classification studies section provides additional examples of how RAT addresses these methodological challenges.

These methodological advantages of RAT open new possibilities for researchers, particularly given the existing challenges of data access. We see great potential for RAT, as research utilizing search results often relies on limited samples collected, evaluated, and analyzed manually. This is particularly true for studies examining results from commercial search engines since researchers usually lack access to such data, as mentioned above.

When scientists cannot obtain a search engine provider’s data, questions arise on the evidential significance of the conclusions acquired using these restricted data collections. These restrictions stem from multiple barriers. First, search engine companies treat their ranking algorithms and result data as core intellectual property and competitive advantages, making them reluctant to share comprehensive datasets that could reveal insights about their systems. Second, even when APIs are available (like Microsoft Bing API), they typically impose rate limits, substantial costs for high-volume access, and restrictions on data storage and redistribution. These barriers particularly impact academic researchers with limited resources compared to commercial entities, making it difficult to conduct large-scale studies that could provide more comprehensive insights into search engine behavior and impact. Such access would help conduct studies that search engine providers do not carry out. These studies could cover information retrieval aspects and areas where the search engine providers are vested in keeping the results hidden because they may contradict their self-interests. The term “self-interest” refers to the primary financial motivations of search engine companies, which can conflict with decisions about relevance or user interests. Self-interest is evident in Google’s tendency to prioritize its content over its competitors (Lewandowski, Sünkler & Schultheiß, 2020). For instance, a competitive investigation by the European Commission led to a record-breaking fine of 2.4 billion euros for Google due to its preference for promoting its shopping results over those of other businesses (European Commission, 2017). Independent studies such as that of (Lewandowski & Sünkler, 2013a) that explore these issues are crucial, as Google is unlikely to make information that contradicts its interests readily accessible (Lewandowski, Sünkler & Schultheiß, 2020).

Apart from addressing the problem of search engine data accessibility, RAT significantly enhances research scalability. The system enables researchers to overcome previous limitations by automating what was once labor-intensive human work, allowing studies to expand from tiny sample sizes to comprehensive datasets. Researchers can define any number of queries and search engines, facilitating more robust and generalizable findings that were previously unattainable through manual methods. The tool automatically queries the selected search engines and grabs the results, removing the bottleneck in manually collecting search results. While automatically collecting results increases the sample size of studies, researchers still need to recruit participants and compensate them for their work if they decide to have results assessed. Even though software cannot remove this barrier, RAT makes assessing results efficient by removing duplicates from the results, providing a user interface for study participants, and storing and summarizing the collected assessments. Study participants can be any individuals recruited by researchers to evaluate search results—from domain experts conducting quality assessments to students performing relevance judgments to crowd workers classifying content. These participants access RAT through a dedicated web-based assessment interface where they can view search results and respond to researcher-defined questions. The interface streamlines the assessment process by presenting results in a standardized format, tracking progress automatically, and ensuring consistent data collection across all participants. Researchers can customize the evaluation criteria and questions based on their study objectives, whether they are conducting relevance assessments, content analysis, or bias detection studies.

Some software tools that at least cover some of RAT’s functionality have been developed in the past. However, these tools have either been developed for a single study only (*e.g.*, Tawileh, Griesbaum & Mandl, 2010; Bar-Ilan & Levene, 2011; Trielli & Diakopoulos, 2020), have not been updated, and therefore have become outdated (Lingnau et al., 2010; Renaud & Azzopardi, 2012), do not offer the flexibility to conduct studies of different types or have been developed for narrowly limited use cases (*e.g.*, Thelwall, 2009; Digitalmethods, 2024). Additionally, proprietary online tools provide scraping capabilities for gathering search results (Oxylabs, 2025; ScraperAPI, 2025). Nevertheless, these services are not open source, which leads to a lack of transparency regarding the technologies and algorithms used and limited configuration options.

To achieve the best possible flexibility, RAT is divided into modules for designing tests, search result scraping, study participant-based evaluations, automatic analysis, and research data download. The scraping module can connect to any web page with a search box, although scrapers must be developed individually for each system (see section Search Engine Scrapers). The scrapers extract structured data from the found search engine result pages (*e.g.*, result descriptions, URLs, position) for further processing. Furthermore, RAT stores the HTML code of all found documents (*i.e.,* search results) and makes screenshots of them for further processing. In addition to the modules mentioned, RAT allows researchers to develop extensions, *e.g.*, for data analysis not covered in the current system.

RAT already has a long history, with the prototype being used in 2012 and referred to as the *Relevance Assessment Tool* (Lewandowski & Sünkler, 2013b). However, the project faced software aging problems that are typical when software is developed and maintained in research projects without particular funding for the software. This situation changed in 2021 when we got funding to develop the new version of RAT from scratch. This redevelopment was required as the field’s technological state of the art had changed.

The funded project covers software development and supports researchers interested in conducting RAT studies. This is especially important as many researchers from non-technical fields are not used to utilizing software like RAT for their research. Another part of the RAT project is building a community of interested researchers and developers.

In this article, we describe RAT in detail. First, we describe the user journey in the software and then explain all modules with the respective technical implementations to show how the journey is realized. Secondly, we show how we handle research data generated in the software and implement software quality assurance. Thirdly, we show the possibilities for RAT in research by presenting possible use cases and examples of realized studies. We conclude the paper with a summary and discussion, including a discussion of the limitations of the software.

## The result assessment tool

RAT is a versatile web-based software toolkit built in Python that uses the PostgreSQL database and Selenium, a software package for conducting automated web browser tests. While study participants use the RAT Assessment Interface to assess copies of search results and answer predetermined questions related to them, researchers can use it to create studies and use classifiers to analyze search results automatically.

The flexible nature of the toolkit enables the execution of practically all types of studies based on search results. In addition to traditional information retrieval (IR) research, classification studies, such as data and qualitative content analyses, are conceivable. RAT consists of several modules that form a toolkit for conducting the abovementioned studies. For example, all available modules are relevant for performing IR studies as they scrape search systems for defined search tasks and search queries. The source texts are then saved, and screenshots are generated, which will be evaluated by study participants in the Assessment Interface. The entire data can then be downloaded and further evaluated. This is a great advantage, as all the procedures required for such studies are available in RAT.

However, the flexibility also allows the conduct of studies that do not require all modules. For example, scraping the systems without storing the source texts and recording ratings would be sufficient to measure the overlapping of search results in search systems or different country versions of the systems. In the case of studies for qualitative content analysis or classification tasks, all modules could be helpful, as the evaluation could also be done directly in RAT if the researchers want to carry out evaluations themselves or if they want to distribute them to other people. RAT also allows uploading lists of URLs not gathered by RAT, but whose results can still be scraped. In addition, the source texts and screenshots can be downloaded for further evaluation. A list of studies that have already been conducted and types of studies that would have benefited from using RAT can be found in the Results section.

A demo of RAT is available at https://rat-software.org/ and is ready to use to design and conduct studies based on search engine data. The demo version is limited to a sample of search engines concerning the number of search queries since a free selection and number of queries could lead to a high expenditure of computing capacity. However, the demo allows researchers to test the software. For access to the full version, researchers can contact the research team, and they will get full access after a review. We also offer the possibility of installing the software toolkit on self-operated servers (https://github.com/rat-software).

In the following, we describe the user journey, the modules and applications, the technologies used, and how these technologies interact with each other and the end users. We also show how we handle research data generated in RAT and ensure the software’s quality.

### User journey in RAT

**Figure 1:**
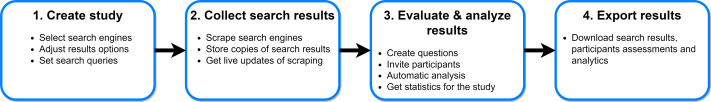
User journey in RAT.

RAT has two user groups: researchers who design studies and study participants who assess web pages. A typical user flow in the software for a study consists of four steps (Fig. 1).

1. **Create study**: The researcher selects the search systems to be analyzed. RAT offers a search engine scraper capable of collecting data from various providers (*e.g.*, Google, Microsoft, DuckDuckGo), including several country-specific versions. This feature supports studies that compare search results across different regions. Additionally, the scraper can extract results from any search system, allowing researchers to specify the types of results they wish to acquire, such as organic search results, snippets, universal search results (*e.g.*, news, images, videos), and advertising. Once the search systems and result types are selected, researchers define the search queries to gather the desired data in the subsequent step.
2. **Collect search results**: The search queries are automatically sent to the selected search system(s). With the help of Selenium, we simulate the search engine calls and save the returned search results. The metadata for each result, such as its position, URL, and description (snippet), is kept in the database. In addition, a copy of the source code and a screenshot of every result document (*i.e.,* the full text of the document, not only the information from the search engine result page) will be created. Live updates on the current status of the scraping process are displayed in the user interface. We have created scrapers for two library search systems; however, additional scrapers may be implemented to facilitate investigations of additional search systems. For instance, scrapers could be implemented to acquire data from social media platforms such as Facebook or X (Twitter), regardless of whether they provide paid access to their data. The benefit of extracting content from the website rather than utilizing the API is that the latter can limit access to specific data. In contrast, the former can be employed to obtain a more significant amount of authentic data. RAT also has the advantage of storing the data content (source code and text) and taking screenshots, which can be used to collect assessments from participants in studies. When scraping search engines or systems, we refrain from using methods that circumvent automated queries’ limitations, such as automatically solving CAPTCHAS. We do not overload search engines or systems with automated queries. We encrypt the screenshots and texts of websites before saving them in the database. We only send individual requests to the websites’ servers to avoid overloading them. We use the information generated during data collection exclusively for research purposes and in no other context.
3. **Evaluate and analyze results**: Researchers can analyze the collected results or create questionnaires to pass on to study participants. For this purpose, researchers can define questions and invite the participants. Study participants evaluate the collected search results on different levels, *e.g.*, assess their relevance to a specific search task or perform classification tasks. For example, a researcher who wants to compare the sources and relevance of Google and Bing results on a particular topic would need to design a test, then use the queries to search for results in both search engines, copy the URLs from the results pages, randomize the URL lists, distribute the URLs to the participants for evaluation, create a list of all domains found in the results, and compare them between the two search engines. To analyze the results, the RAT calculates statistics that can be displayed in the user interface. RAT also offers the possibility of using self-defined classifiers to analyze search results automatically. A search engine optimization (SEO) classifier is implemented at this point. SEO is “all measures suitable for improving the position of Web pages in search engine rankings” (Lewandowski, 2023, p. 175). The SEO classifier calculates the probability of using SEO for a specific URL. It is possible to add further classifiers using the templates provided by RAT.
4. **Export results**: RAT allows researchers to download and process the results of the scraping processes, analyses, and evaluations by study participants at any time. For this purpose, the data is exported from the database and made available as downloadable CSV files.

### Modular structure of RAT

**Figure 2:**
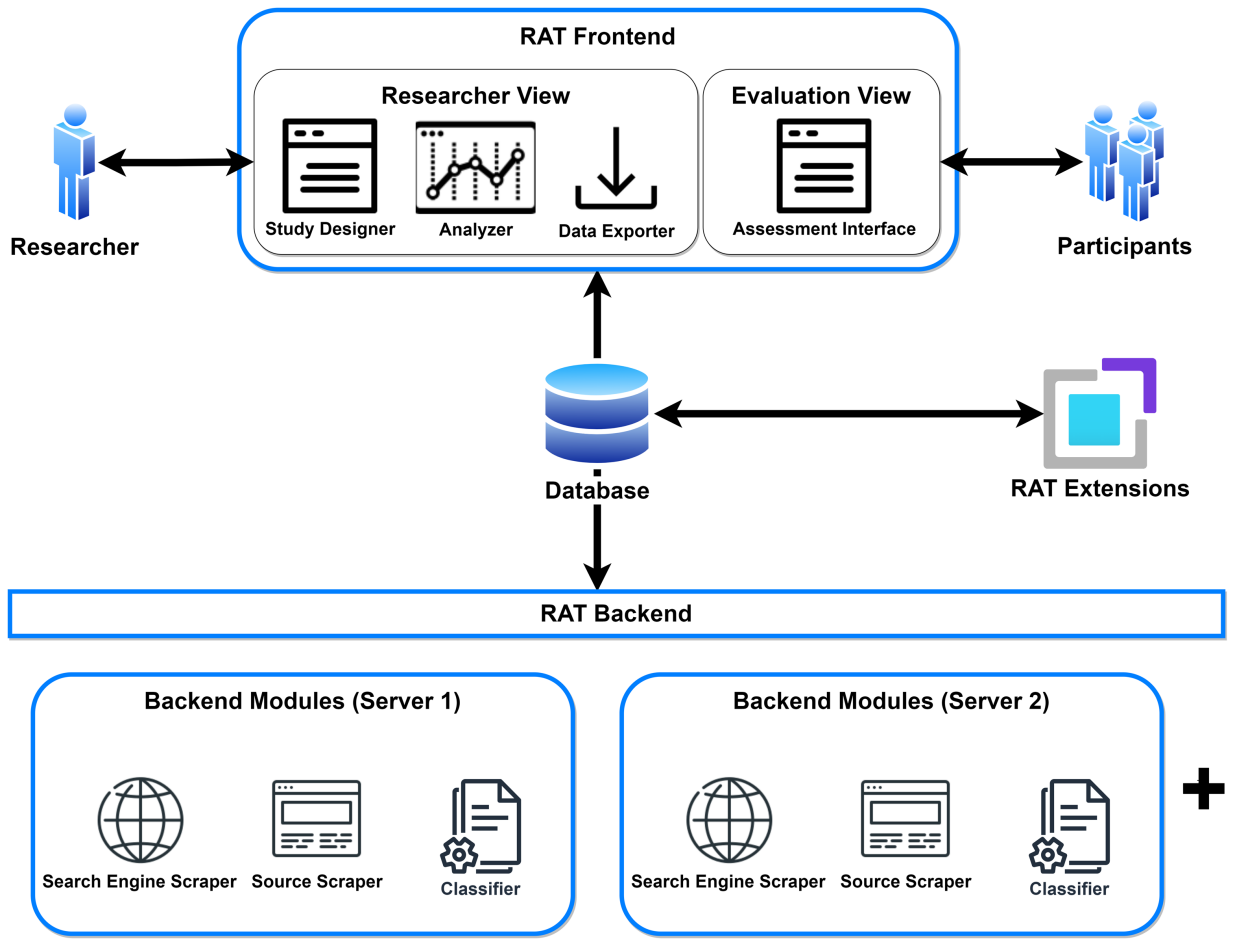
Overview of the applications and their modules in RAT.

The software is divided into two applications that can be installed on different computers and are connected *via* the PostgreSQL database. This approach allows researchers to share resources from time-consuming and computationally intensive processes. One application is RAT Backend, which provides the scraping processes and classification tasks; the other is RAT Frontend, which serves as a graphical user interface for researchers to design studies and for study participants to evaluate search results. We also offer an infrastructure and a dedicated repository (https://github.com/rat-extensions/) for developers and researchers to build extensions for RAT using the data provided through the database. The overview of the software is shown in Fig. 2.

### RAT Frontend

**Figure 3:**
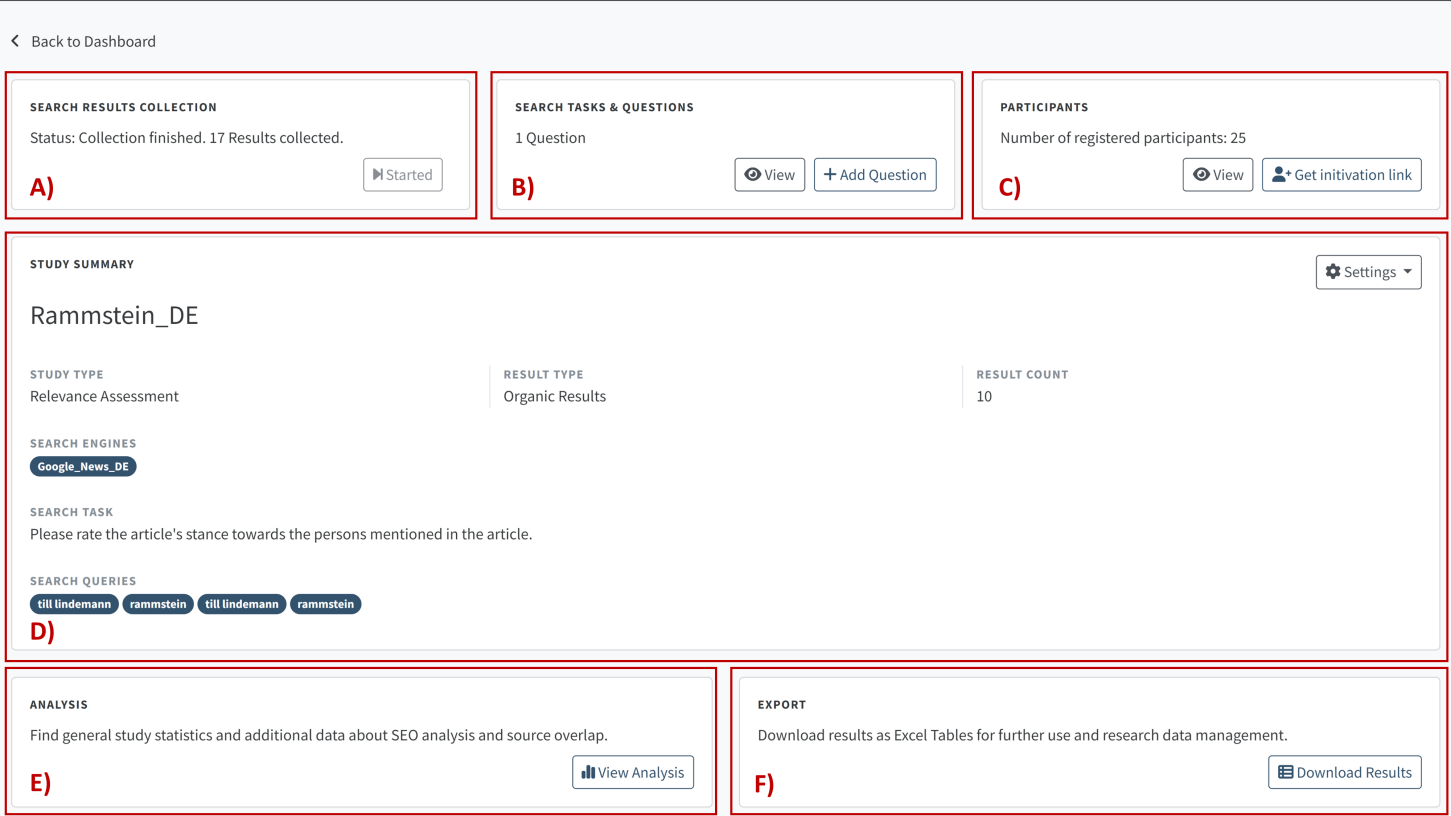
Researcher view in RAT.

**Figure 4:**
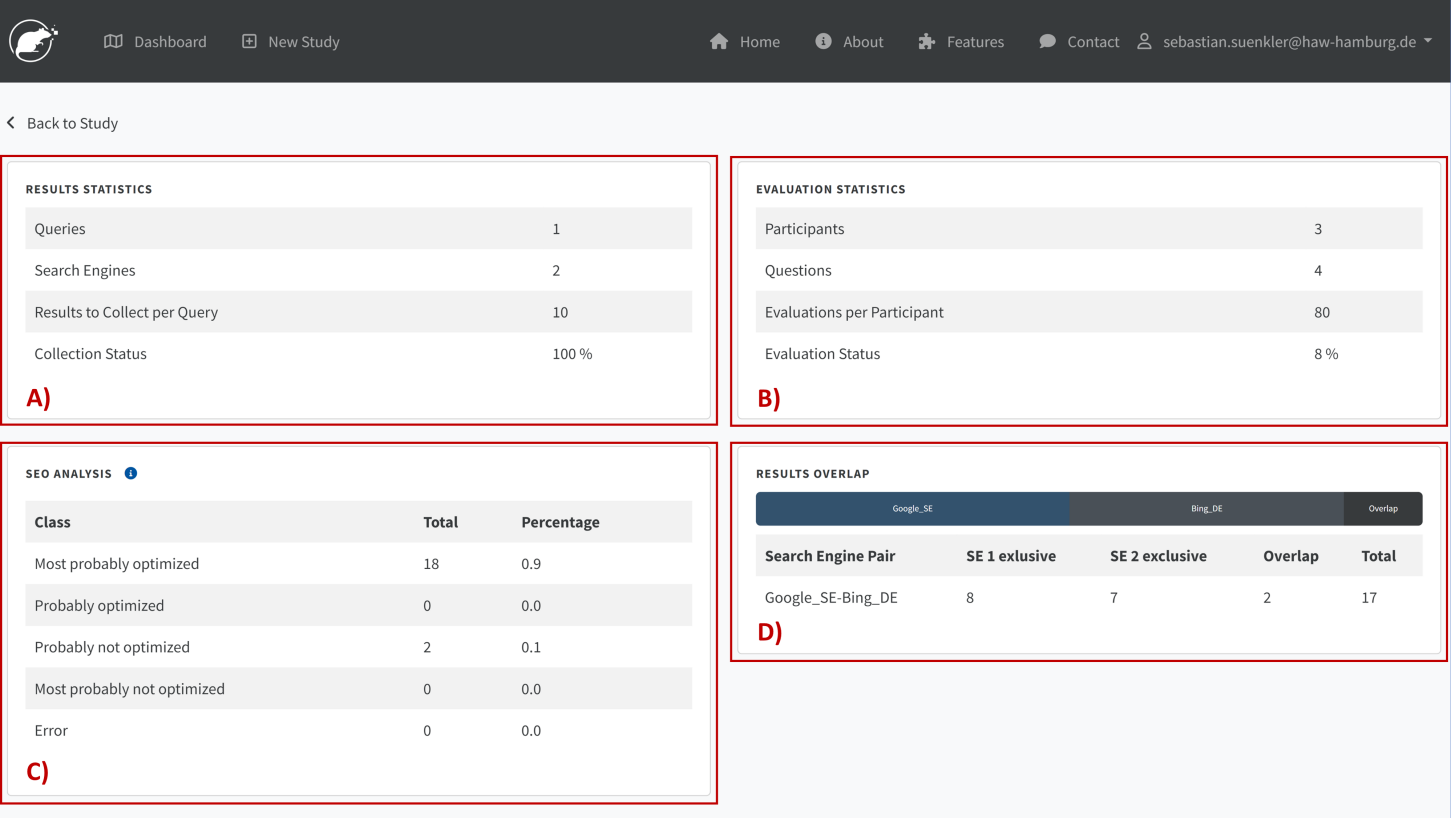
Results and statistics from the analyzer module in RAT.

**Figure 5:**
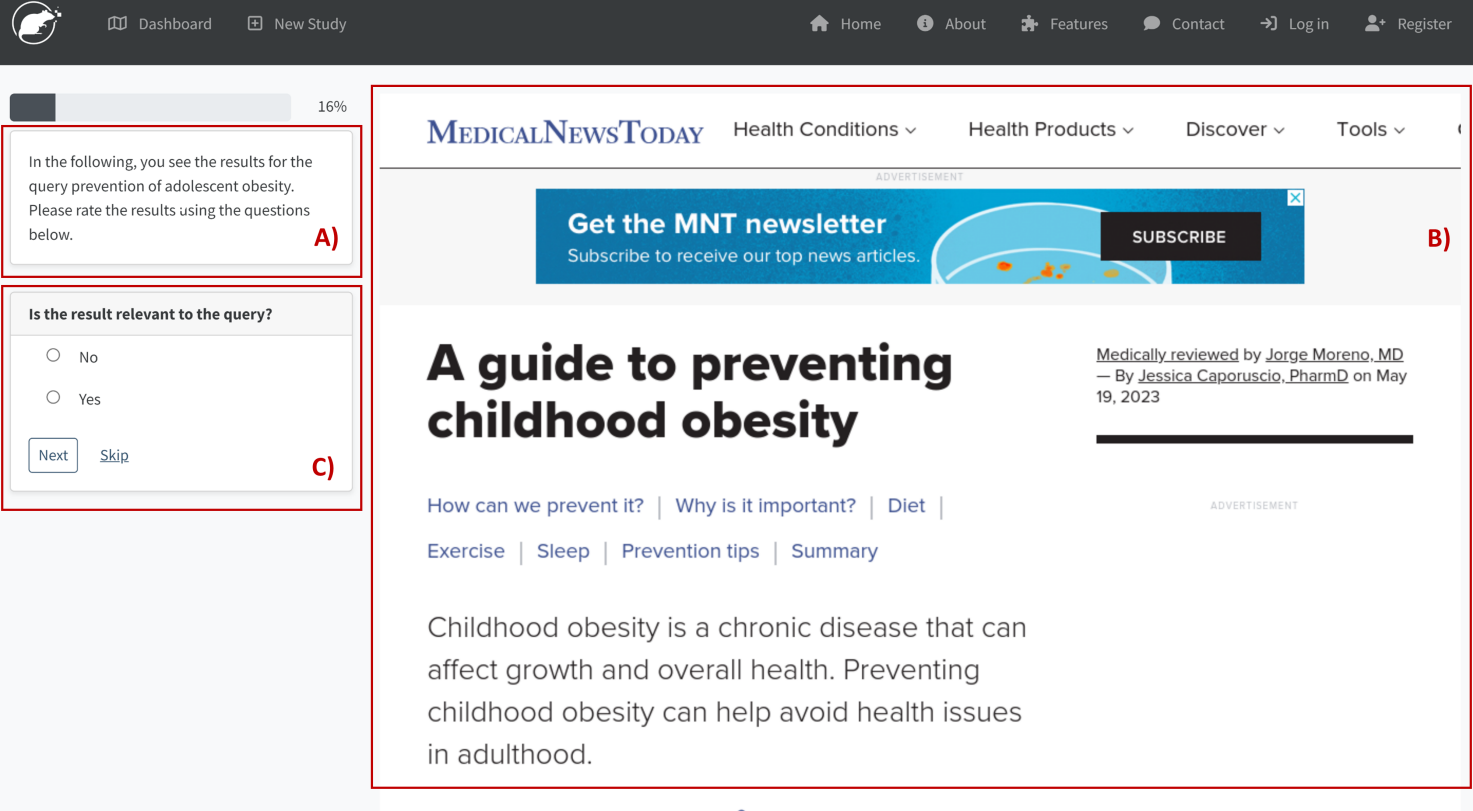
Evaluation view in RAT.

RAT Frontend is developed in Flask, a micro-web framework written in Python, and it is the GUI of RAT. We use Flask because of its functionality and flexibility in meeting our software requirements. These requirements include a secure, lightweight application framework that can scale up to complex applications. Its architecture also supports the development of controllers and views, making the source code more sustainable and extending the software easily. We also use several Flask extensions, like Flask-SQLAlchemy (support for SQLAlchemy to connect to the PostgreSQL Database), Flask-Login (user session management), Flask-Mail (interface to set up SMTP), and Flask-WTF (integration with WTForms for secure forms with CSRF token and file upload management) which offer all the basic functionalities of usable and secure web-based software. Researchers and study participants interact with RAT *via* RAT Frontend, which includes a Researcher View (Fig. 3) for designing studies and analyzing study results (Fig. 4) as well as an Evaluation View (Fig. 5) for collecting study participants’ assessments or assessments by researchers.

### Researcher view

Researcher View gives access to Study Designer, Analyzer, and Data Exporter through a dashboard. Study Designer is the basic module researchers use to specify the study parameters, search tasks, search queries, search engines, search result options, and manage study participant access.

In Researcher View, researchers also define the questions that will be asked in Assessment Interface. Researchers launch the search engine scraper in RAT Backend here (Fig. 3A) and receive real-time information about the scraping progress. Regarding question design, RAT is quite adaptable (Fig. 3B). Open-ended questions, Likert scales, sliders, and multiple-choice questions are examples of question types. The questions are presented using Bootstrap (https://getbootstrap.com/docs/4.0/components/forms/). Bootstrap is an open-source CSS framework for creating responsive web applications. Bootstrap provides templates with classes for forms to display all question types available in RAT. Such questions could include, for example, “How relevant is the result shown here?”; “Would you see this result coming from a reputable source?”; and “Is this text well-written?” In this view, the participants are also managed (Fig. 3C). Figure 3D shows an overview including the selected search engines, defined search queries, and the study type. All these options can be changed if scraping has not yet begun.

Analyzer (Fig. 3E) and Data Exporter (Fig. 3F) are additional modules in Researcher View. Analyzer provides tools for automatically analyzing search results. Figure 4 shows some examples of processed data. In addition to statistics on the scraping progress (Fig. 4A), it also provides information on the evaluation process (Fig. 4B). These statistics are essential for tracking the study’s progress. The figure also shows an example of an implemented classifier that determines the probability of SEO on the search results collected (Fig. 4C). Another standard analysis measures the overlap between the results from different search engines in a study (Fig. 4D). Open-ended questions, Likert scales, sliders, and multiple-choice questions are examples of question types.

### Evaluation view

Another view in RAT Frontend is the Evaluation View (Fig. 5). Participants register through a link provided by the researchers. This process creates credentials for the study participants so they can log in to work on the given tasks. Another scenario is the researchers creating credentials to evaluate or classify the collected search results. In general, this approach allows anonymous access for participation in studies. We also do not collect data that makes participants identifiable. However, researchers could send the link to registration to individuals, *e.g.*, to conduct group comparisons or distribute incentives.

In Assessment Interface, study participants or researchers navigate through the copies of the search results related to the search task (Fig. 5A). To ensure clarity, the term “participants” will refer to external participants and researchers conducting the assessments themselves in the following text. Respondents reply to the defined questions (Fig. 5B) using a screenshot of the search result or an uploaded document (Fig. 5C). The procedure in the Assessment Interface is identical for each evaluator. The participant first uses their previously generated individual login credentials to log in, after which they see a questionnaire with a saved screenshot of a search result. The questionnaire is made up of the questions that were previously defined in the Study Designer. The reviewer can now answer these questions or skip the assessment if there are uncertainties or display errors. A progress bar provides information on how far the assessments have already progressed. Participants also have the option of pausing the assessments at any time and resuming them at a later date. When designing the interface, we prioritized ease of use and followed standard online survey conventions to ensure straightforward access. Researchers who wish to use this interface to collect ratings from external study participants can also directly enter a procedure description. The database stores the answers, enabling investigators to access them through Data Exporter (Fig. 3E) and download the participant assessments. Data Exporter produces multiple Excel tables, which the research team can utilize for additional analysis. In addition to the assessments, the table includes a list of gathered search results and predetermined questions.

## RAT Backend

The other application of RAT is RAT Backend, which utilizes the database to receive and process the inputs from Researcher View in RAT Frontend. It is a collection of modules to (1) collect search results (Search Engine Scraper), (2) extract the source code and take screenshots of the search results (Source Scraper), and (3) a framework to add classifiers to RAT (Classifier) for automatic analysis processes based on the search results. In contrast to the RAT Frontend, no graphical user interface is available; all configurations are realized through JSON files.

**Figure 6:**
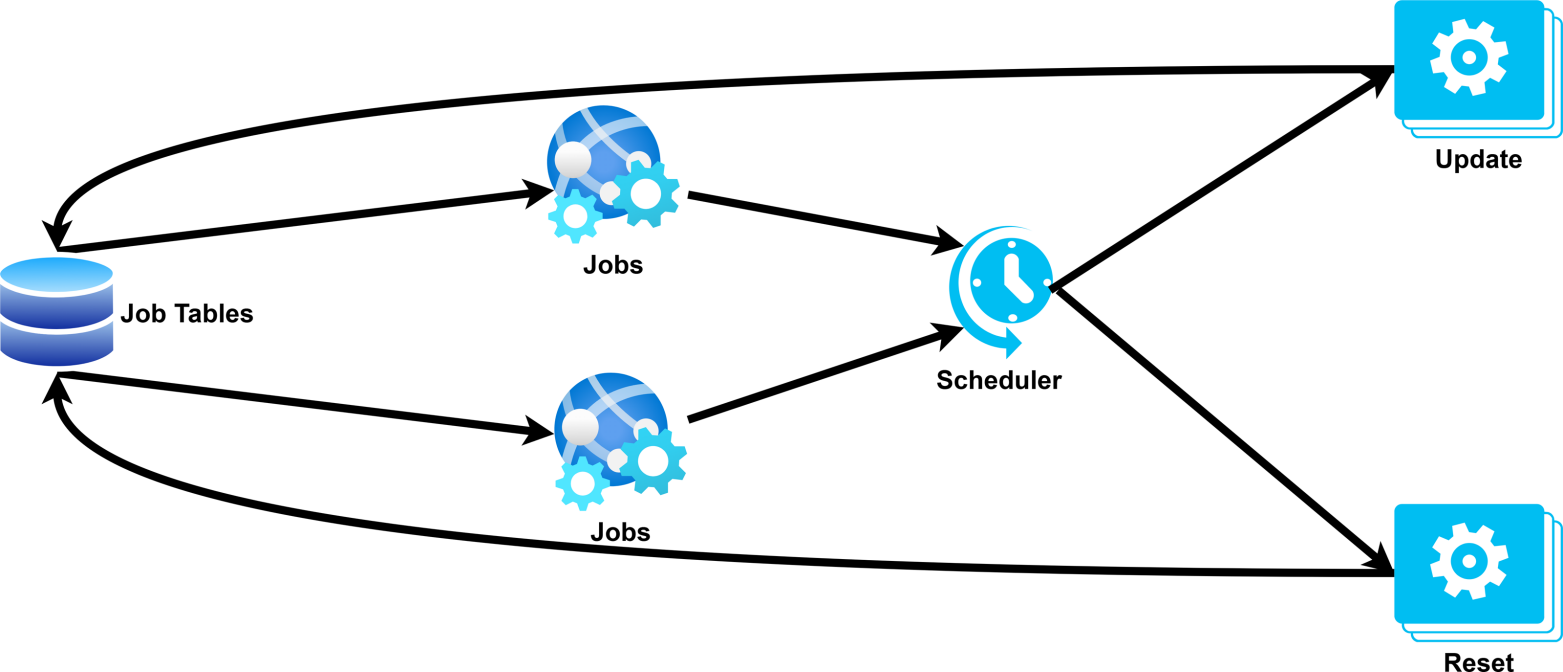
Scheduler and job management in RAT.

The core architecture of RAT Backend is built on an integrated job management system utilizing the Advanced Python Scheduler (APScheduler) library. Figure 6 illustrates the job management process in RAT.

This system coordinates all automated processes through controllers that handle the scraping of search results, storage of source code and screenshots, and classification tasks based on configuration files and researcher input from the RAT Frontend. Each module works with special request tables in the database that create processing dependencies: Unprocessed search queries trigger the scraping process of the search system, which then activates the source scraper as soon as the results are saved and finally initiates classification as soon as content is available. With built-in error handling that resets and reschedules unsuccessful jobs, the system automatically plans, executes, and tracks job status. Regardless of the precise tasks being carried out, this architecture guarantees effective resource allocation while preserving a consistent workflow across all modules.

### Search engine scrapers

In RAT, research is conducted using search results collected through an automated scraper. Once implemented, this system allows researchers to define search queries and select various search engines (*e.g.*, Google, Microsoft Bing) or any web-accessible search system, including social media platforms like X (Twitter) and Facebook, as well as on-page search functionalities (*e.g.*, https://www.haw-hamburg.de/en/search/). Unlike traditional APIs, our scraper mimics a user’s actions in a browser, which allows for fewer restrictions on the data collected. We selected our scrapers based on the popularity of commercial search engines and tested Google and Bing against alternatives such as Ecosia and DuckDuckGo. In addition, our software is designed to be flexible, allowing researchers to add custom scrapers tailored to their specific studies. This flexibility ensures that the current selection of search engine scrapers does not limit research.

**Figure 7:**
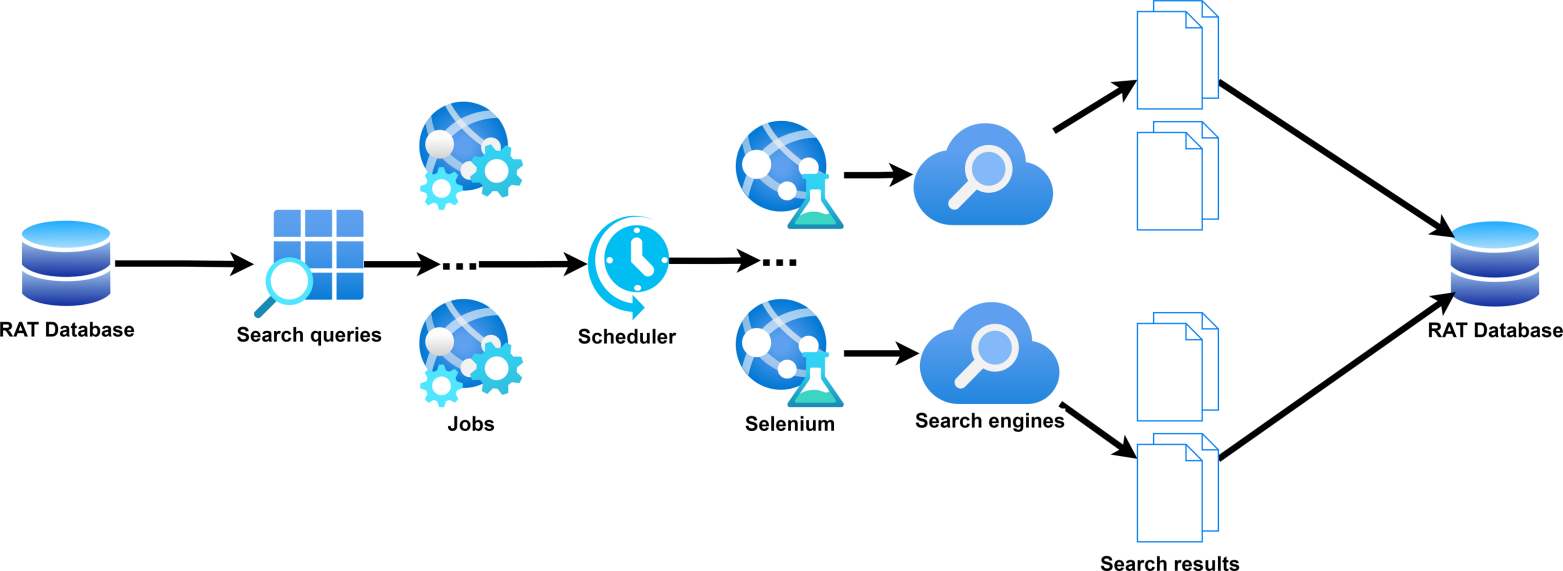
Search engine scraper in RAT.

Search Engine Scraper (Fig. 7) is a crucial module in RAT Backend that is responsible for the automatic collection of search results.

The module creates jobs from the queries and search engines selected to collect results from these engines and writes them into a job table. A scheduler regularly checks for open jobs and starts Selenium, a suite to automate tests for web applications. Its capabilities also allow the simulation of browser interactions with any URL by providing web drivers for all major browsers (https://www.selenium.dev/documentation/webdriver/). When searching, we can simulate user input to send the query, allowing us to obtain the search engine result page (SERP). The module reads the page content and uses a parser to extract the search results by identifying specific CSS selectors for the title, description, and URLs. Since every search engine and retrieval system employs different styles for their SERPs, it is necessary to identify the relevant CSS selectors individually. Additionally, scrapers must be adjusted to collect results beyond the first page. This can be done by extracting links from the pagination or simulating interactions, such as scrolling down when the search system uses continuous scrolling to display more results. We also provide a script to add the new scraper to the database (https://github.com/rat-software/rat-software/blob/main/templates/add_scraper_to_database.ipynb) Usually, the parameters listed in Table 1 need to be defined for a scraper in RAT.

**Table 1:** Parameters for search engine scraper in RAT.

Parameter	Description	For instance (Google Germany)
SEARCH_URL	URL of the search engine with optional GET-parameters provided by the search engine (*e.g.*, to set a language parameter)	https://www.google.de/webhp?hl=de
SEARCH_BOX	Input element in a form to enter a search query.	<textarea class=“gLFyf” name=“q” </textarea>
CAPTCHA	Source code hint to find a CAPTCHA element.	g-recaptcha
NEXT_PAGE	CSS element for a click button to navigate to the next search engine result page (SERP)	//a[@aria-label=‘{}’]
RESULTS_NUMBER	Parameter to initialize the number of the first result.	0
PAGE	Parameter for the first SERP.	0
CSS_RESULT	CSS class for each div container with a result consisting of a title, description, and URL.	“div”, class_=[“tF2Cxc”]
CSS_TITLE	CSS class of header for a title in a result container.	“h3”, class_=[“LC20lb MBeuO DKV0Md”]
CSS_DESCRIPTION	CSS class for the description. A description is part of the search result.	“div”, class_=[“VwiC3b”]
CSS_URL	CSS class for the URL in a result container.	“h3”, class_=[“LC20lb MBeuO DKV0Md”]

However, there are exceptions, as search engines may deliver search results in non-standard formats. For instance, results from a library system might include additional metadata, such as book authors, publisher information, and more, beyond the typical title, description, and URL. This additional information must be summarized and stored in the standard description column within the table used for storing scraped search results.

Each scraper needs its own script in order to function. We offer a template script for creating new scrapers (https://github.com/rat-software/rat-software/blob/main/backend/scraper/scrapers/template_new_scraper.py) and Jupyter Notebook (https://github.com/rat-software/rat-software/blob/main/templates/new_scraper.ipynb) for on-the-fly development and testing. It is also important to note that RAT was developed specifically for HTML-based web interfaces and is not suitable for non-web-based search systems.

Search engine scrapers need to be updated frequently, as search engine operators regularly change the format of their SERPs or their methods of delivering search results, such as using pagination or continuous scrolling. To address this, RAT provides a daily job to test the functionality of the implemented scrapers. If a scraper test fails, the scraper is stored in the database and marked as unavailable for further studies. Researchers who developed the scrapers are notified *via* email when their scraper stops working, prompting them to fix the issue. This notification indicates that something, such as the HTML or CSS code on a SERP, has changed, requiring the scraper to be updated accordingly. Testing all scrapers before beginning a new study is highly recommended to ensure their functionality.

Apart from structural modifications to SERPs, a major difficulty in large-scale search engine research is negotiating the several protective policies put in place by search engines. Most search engines block automated access by means of anti-bot policies and rate limits. Search engine operators may momentarily block the scraper’s IP address if they notice several requests from the same server, therefore failing jobs. RAT solves this problem using various technical tools. First, the system distributes requests over time to avoid activating rate limit protections by automatically resetting and restarting failed jobs after a configurable waiting period. RAT also enables proxy server integration so researchers may set up rotating proxy lists spreading requests over several IP addresses. This greatly raises the amount of data that can be gathered before running into blocks.

Although these policies greatly enhance data collecting capacity, search engines always change their detection techniques, and sometimes finding and blocking even advanced methods using Selenium. RAT keeps thorough error codes for failed jobs in the database to preserve research integrity, therefore giving researchers the knowledge required to grasp collection constraints and modify their approach as appropriate. Moreover, RAT’s distributed architecture lets several instances run concurrently while coordinating *via* the centralized job management system, so allowing large-scale data collecting activities to be spread across several networks and geographic areas.

With respect to CAPTCHAs, we use ethical scraping techniques. The system records the occurrence of a CAPTCHA and puts the job back in the queue for a later retry instead of trying to bypass these safeguards. Jobs that regularly set off CAPTCHAs are marked for researcher review since this could suggest a need to change collection parameters or timing. We follow reasonable rates of collection that honor search engine resources and use adjustable delays between requests. Even with these protections, we understand that search engines might put fresh anti-scraping policies that might compromise collection completeness. RAT records all collection problems to keep openness regarding these constraints, therefore enabling academics to record and compensate for any systematic deficiencies. By respecting the search engine’s protective measures, this strategy preserves ethical integrity; by correctly recording collection limits instead of presenting incomplete data as complete, it preserves methodological integrity.

### Source scraper

Source Scraper is the module responsible for fetching the content of search result documents or submitted URLs and storing the source code and screenshots in the database. We use Selenium WebDriver for this task because it provides the necessary functionalities to interact with a web page (*e.g.*, scrolling) and execute JavaScript, which is crucial for gathering dynamic content. These capabilities are essential for two reasons. First, we want study participants to evaluate documents in the same format they would receive when using a web browser. Second, we need to capture the entire HTML source code, including all content, for classification purposes. For instance, the SEO classifier requires all HTML tags, and if some texts are only rendered by scrolling, these must be captured to accurately assess the web page’s readability.

Other web parsers, such as BeautifulSoup or cURL, are not suitable for this task because they do not support server-side rendering and cannot fetch all content from a web page. Additionally, the module takes screenshots of the URLs, which are made available to study participants in Assessment Interface. This process occurs at a specific time, ensuring that study participants always see the content as it was when scraped, preventing issues such as 404 errors or viewing documents that have been modified since the study was designed and the data collected.

Source Scraper employs the same job management architecture as other RAT modules to systematically collect and process content. The system anticipates various failure scenarios, such as temporarily inaccessible URLs, and implements automatic retry mechanisms scheduled at configurable intervals. To minimize potential biases, we have incorporated several features. The proxy architecture established for search engine scraping extends to source collection, ensuring content is fetched from the appropriate geographic region to match the country version of the search engine being studied. This prevents the inadvertent collection of automatically translated versions of websites that could introduce language bias into the research data. Furthermore, we utilize the browser extension “I Still Don’t Care About Cookies” (https://github.com/OhMyGuus/I-Still-Dont-Care-About-Cookies) to bypass cookie consent banners and notifications that might otherwise obscure important content during capture. The system also automatically simulates scrolling behavior to ensure a comprehensive collection of lazy-loaded content that only appears as users navigate down a page. Together, these measures help ensure that collected data accurately represents what real users would encounter, addressing potential sampling biases that could arise from incomplete or geographically inconsistent content collection.

### Classifier

Using the classification module allows for automatic classifications based on the data collected by RAT. The classification is always executed on a search result or a URL uploaded to RAT. RAT allows for adding any classifiers using templates and the database. Templates and examples such as Jupyter Notebooks are available at https://github.com/rat-software/rat-software/tree/main/templates (README.MD includes a tutorial on how to use the notebooks). After testing classifiers, they can be added to the database by using the script provided. All classifiers can use the data RAT currently provides:

• **URL** of a web page
• **Domain** of the web page
• **Position** in search engine ranking
• **Title** from the search result snippet
• **Description** from the search result snippet
• **IP address** of a web page
• **Source code** from a web page
• **Screenshot** of a web page
• **Search query** from which the result originates

The classification result from any classifier will be stored in the database as key-value pairs concerning the search result. One classifier already available in RAT is the SEO classifier to estimate the probability that has been used on a website (Lewandowski, Sünkler & Yagci, 2021) (https://github.com/rat-software/rat-software/tree/main/backend/classifier/classifiers/seo_rule_based).

The system automatically retries failed classification jobs up to a maximum number of attempts, which the researcher can configure. Researchers can set custom parameters for each automated classification job that define when a job should be considered failed. The classification process is determined by the classifiers assigned to a study—the system identifies these assigned classifiers and initiates the corresponding jobs. All classifiers use the provided information in the database, like the stored content of a web page, screenshots, or metadata about collected search results.

### RAT extensions

We have already mentioned some possibilities for extending RAT by adding new search engine scrapers and classifiers. However, RAT is also extendable by developing software that utilizes the RAT Database. These add-ons include a web browser plugins such as the Explicit and Implicit Logger (EI Logger) for determining the use of search engines for conducting interactive information retrieval (IIR) studies, a scraper for extracting contact data from content providers’ legal notice pages, and a tool for calculating a readability score for the text of a web page. All add-ons are connected to the RAT database and tables, so that the data generated from the extensions can be added (https://github.com/rat-extensions/). Table 2 gives details on some of these extensions.

**Table 2:** RAT extensions.

Extension	Programming languages	Description	Availability
Imprint crawler	Java	A web crawler that is able to automatically extract legal notice information from websites while taking German legal aspects into account.	https://github.com/rat-extensions/imprint-crawler
Readability score	Python	A Python tool that extracts the main text content of a web document and analyzes its readability. Input data: URL or/ Search Query. Output data: For a URL, the output includes the detected language and the readability score, calculated using different formulae, along with the average reading time.	https://github.com/rat-extensions/readability-score
Forum scraper	Python	An extension to extract comments from German online news services.	https://github.com/rat-extensions/forum-scraper
EI logger	Typescript, Java	A browser extension for conducting interactive information retrieval studies. With this extension, study participants can work on search tasks with search engines of their choice and both the search queries and the clicks on search results are saved.	https://github.com/rat-extensions/EI_Logger_BA
Identifying affiliate links in web pages	Python	A Python tool that extracts all affiliate links of a web document and scores this web page according to its number and prominence of affiliate links.	https://github.com/rat-extensions/Identifying-affiliate-links-in-webpages
App reviews scraper	Python	These app scrapes reviews, that will visit designated URLs of a set of applications and export the scraped reviews and relevant information.	https://github.com/rat-extensions/app-reviews-scraper
Visualizations of IR measures	Python	This add-on aids researchers to have some initial visualizations based on the standard IR evaluation measures. There is a config.toml file for the theme.	https://github.com/rat-extensions/ir-evaluation
Scraping news articles	Python	This Python tool retrieves the homepages of given news portals and scrapes the HTML text of the articles found. Each text is saved in a separate file. For each portal, an overview file is created, which contains the metadata of the articles and the corresponding file paths.	https://github.com/rat-extensions/NewsArticlesScraper

### Handling of research data generated by RAT

RAT generates data by scraping search results (*i.e.,* copies of web pages in HTML form and as screenshots), collecting participant evaluations (*i.e.,* answers to questionnaire items), and computing indicators, classification results, and scores in automatic analysis. Data management plans (Rat für Sozial- und Wirtschaftsdaten, 2018) are prepared to describe the research data comprehensibly, explaining details such as the software and version numbers required for reusing the data. We also follow the Findable, Accessible, Interoperable, Reusable (FAIR) guiding principles for scientific data management and stewardship (Chue Hong et al., 2021). Furthermore, since RAT is research software, we adhere to the FAIR principles for research software (FAIR4RS Principles; Chue Hong et al., 2021), a revised and extended version of the general FAIR principles. We follow the FAIR4RS principles, as detailed in Table 3.

**Table 3:** FAIR4RS for RAT.

**Findable: Software, and its associated metadata, is easy for both humans and machines to find.**
*F1: Software is assigned a globally unique and persistent identifier:* We use Zenodo to create unique and persistent DOIs for our releases in GitHub. Zenodo offers an integration of GitHub to create DOIs automatically when a release is published.
*F2: Software is described with rich metadata:* We use the citation file format CITATION.cff in our GitHub repository for describing the software with rich metadata.
*F3: Metadata clearly and explicitly include the identifier of the software they describe:* CITATION.cff has a field for including the identifiers of the releases clearly and explictitly.
*F4: Metadata are FAIR, searchable and indexable:* The use of Zenodo and CITATION.cff ensures that the metadata are FAIR, searchable and indexable.
**Accessible: Software, and its metadata, is retrievable via standardized protocols.**
*A1: Software is retrievable by its identifier using a standardized communications protocol:* The software is retrievable by its persistent identifier from Zenodo and the URL to the GitHub repository. This allows researchers to download and install the software on a (local) webserver.
*A2: Metadata are accessible, even when the software is no longer available:* The software is accessible on several websites and repositories over https. While the software and source code are available without further authorization, using the software as a service will be possible through a closed authentication procedure. We ensure that metadata will remain accessible by sharing it on Zenodo and using CITATION.cff.
**Interoperable: Software interoperates with other software by exchanging data and/or metadata, and/or through interaction via application programming interfaces (APIs), described through standards.**
*I1: Software reads, writes and exchanges data in a way that meets domain-relevant community standards:* Domain-relevant community standards cannot be applied to the software because we use a specific schema to store the result types in RAT (search results, results from the automatic analysis and classifications). However, we integrated search engine APIs, *e.g.*, Microsoft Bing and Google Keyword Planner, to retrieve results from these services. We will write extensive documentation how to use these APIs in RAT. We also plan to develop a RAT-API for researchers, and we will evaluate the *FAIRsharing* standards to meet relevant community standards.
*I2: Software includes qualified references to other objects:* There are several qualified references to external data objects required to execute the software. The most important qualifiers are that RAT is implemented using Python and uses PostgreSQL.
**Reusable: Software is both usable (can be executed) and reusable (can be understood, modified, built upon, or incorporated into other software).**
*R1: Software is described with a plurality of accurate and relevant attributes:* We decided to publish the software under the GPL-3.0 license to give it a clear and accessible license. As for the provenance, we added descriptions to our metadata about the intent and origin behind the software development.
*R2: Software includes qualified references to other software:* There are several qualified references to other software. The most important is using Selenium Project to scrape search systems and web content.
*R3: Software meets domain-relevant community standards:* We decided to use a popular and regularly maintained programming language (Python) and database (PostgreSQL) to develop the software. The decision to use Selenium as the main program for scraping web content was made because it is the de facto standard for automated web testing and has a large developer community. We also plan to offer parts of RAT as packages via PyPI, as this is the community standard for package management in Python.

In addition, regular plausibility checks further ensure data quality during the data collection phases. We make all non-personal research data we generate when conducting studies in the research group permanently available. The data is available to other researchers *via* the Open Science Framework (OSF) (https://osf.io/t3hg9/). All software components developed in the project are available *via* GitHub (https://github.com/rat-software/). No retention periods exist, so the data is made available immediately after collection and processing (*e.g.*, pseudonymization). In addition, we support and advise researchers who use the software in their research to publish the data they generate with RAT.

### Software quality assurance

We ensure software quality through both technical and human-centered testing approaches. On the technical side, we have built a software quality assurance framework that involves regular testing with pytest for all RAT modules. We routinely run automated tests for the search engine scrapers, classifiers, and frontend to identify technical issues and bugs before they affect real research.

On the human side of testing approaches, we develop RAT according to the user-centered design (UCD) principles (International Organization for Standardization, 2010, p. 11). A crucial component of the UCD process is the evaluation of the design. These evaluations include tests of paper prototypes and mockups, heuristic evaluations, and usability tests. Subjects for the usability tests are researchers from the different user groups, as mentioned in the results section below. Subjects participate in the lab or remotely, depending on their accessibility. The entire development process is accompanied by evaluations of the design, with the results of the evaluations being used to increase the usability of RAT.

In addition to design evaluations, RAT provides a feedback management system. This feature allows RAT users to provide input while using the software, helping to uncover technical and logical software errors. The feedback system has been actively used by researchers and students conducting studies with RAT. Their feedback primarily highlighted technical errors in data export and scraping and suggestions for enhancing the software’s frontend. One of the examples is that we gathered ideas for improving the frontend, such as adding a button for study participants to return to a previously evaluated result and ensuring the evaluation question remains fixed while scrolling in the Assessment Interface.

Another significant example of how our quality assurance process improved data collection reliability involved the Google scraper. We discovered an issue where the scraper returned fewer results than specified—*e.g.*, scraping only 24 results when the limit was set to 30. While search engines commonly return fewer results than requested for various legitimate reasons (such as query specificity, limited matching content, algorithms, filters, or rate limits), our investigation revealed a technical bug in the Google scraper that artificially limits results across queries regardless of content availability.

The bug was initially identified through systematic data validation. We observed inconsistent result counts across similar queries and noticed that this pattern occurred unpredictably across query types rather than correlating with query content or complexity. To isolate the issue, we ran the scraper in non-headless mode (with a visible browser interface) and observed its behavior directly. This revealed that our scraper was failing to properly handle Google’s dynamic loading mechanism, where additional results are loaded as users scroll down the page rather than through traditional pagination links that our scraper was designed to detect.

This premature termination of the scraping process raised concerns about potential research implications. The artificial limitation could introduce systematic bias if specific results typically appear lower in the ranking positions. In comparative studies, having fewer data points than planned could reduce the statistical significance of findings. Additionally, when comparing results across different search engines, the bug could create inconsistencies where one engine returns the complete requested set while another is artificially limited.

After fixing the bug by implementing detection for both pagination and infinite scrolling result presentation methods, we validated the solution through several measures:

1. We retested the same queries that previously showed limited results to confirm that the scraper now retrieved the expected number of results.

2. We implemented automated validation tests that verify result counts against expected thresholds.

3. We added logging to record result count discrepancies for researcher review specifically.

Beyond this specific issue, we continuously monitor for other potential biases in data collection. Search engines frequently modify their algorithms and interface elements, affecting scraping reliability. To address this challenge, our daily automated scraper tests detect when search providers change their SERP structure, allowing us to update scrapers promptly. We also track search engine algorithm updates through industry resources, verify our collection methods after significant changes, and implement geographic diversity through our proxy configuration to prevent region-specific biases.

From a methodological perspective, we ensure integrity by documenting all collection parameters, including rate limits, timeouts, and error-handling procedures, so researchers can fully disclose these details in their publications. This documentation is critical for research replicability and understanding potential systemic biases in the collected data.

## Results

In this section, we outline RAT use cases with studies that have already been conducted using the software and studies that could have benefited from it. We searched literature to identify potential use cases and understand the use context, a fundamental step in the user-centered design (UCD) process (International Organization for Standardization, 2010, p. 11).

In Scopus (https://www.scopus.com/), we searched for studies published between 2016 and 2021 in which search results were evaluated (evaluate, rate, assess, or other synonyms). We limited the literature search to studies using data from commercial search engines and library search systems. RAT was explicitly designed to analyze data from web-based search interfaces that provide ranked results to user queries. This focus stems from the challenges researchers face when studying these systems: unlike social media platforms or specialized databases that often provide APIs, major search engines rarely offer systematic access to their results data.

RAT addresses this gap by providing tools to systematically collect, store, and analyze results from any web-based search interface. By “systematic collection”, we mean that RAT employs a structured approach to gathering search data beyond simple HTML scraping. The system captures three key data types: (1) metadata from search engine result pages (including titles, descriptions, URLs, and ranking positions), (2) the complete HTML source code of both the SERP and each result document, and (3) visual screenshots of how each page appears to users. This comprehensive collection enables researchers to analyze what results are returned, how they are presented, and what content they contain.

For example, when analyzing information across search engines, a researcher could use RAT to simultaneously collect results from Google, Bing, and specialized search systems for identical queries, store normalized data in consistent formats, capture the full text and visual presentation of each result page, and then systematically assess these results using either human evaluators or automated classifiers. This structured approach ensures that all data points are collected using identical parameters and stored in standardized formats, enabling valid cross-system comparisons.

We first screened the publications by study type and discipline based on the abstract. Then we checked the paper for methodological details, such as the search engines and the number of queries employed for result assessment. We did not conduct a systematic literature review but synthesized different RAT use cases, checking results until no fundamentally new use case could be identified. This approach resulted in a thorough evaluation of 36 publications describing studies that RAT could have supported.

Our literature review revealed that traditional approaches to search engine research face significant limitations. Researchers typically collect search results manually by copying and pasting from search engine result pages or using basic web scraping scripts that need frequent maintenance. This process is time-consuming, error-prone, and severely limits sample sizes. Studies in our review were limited to analyzing fewer than 100 queries due to constraints of human-conducted data collection

RAT addresses these limitations through automated collection and systematic processing. While automation introduces its challenges—such as navigating anti-bot measures and ensuring consistent data quality—RAT implements specific technical solutions like proxy rotation and automatic error recovery to maintain data integrity. Where traditional methods might take weeks to collect a few hundred results, RAT can gather thousands of results in hours while capturing screenshots and source codes for future reference. For instance, Yagci et al. (2022) were able to analyze over 141,000 results from multiple search engines—a scale that would be impractical with traditional methods. While large datasets are valuable for many research questions, RAT’s flexibility also supports smaller-scale studies requiring more focused qualitative analysis.

The following presents RAT use cases, covering studies that have already been conducted with RAT and studies that could have been supported by RAT, as identified in the literature search. In Table 4, we summarize the studies. In the next sections, we describe the studies grouped by study type.

**Table 4:** RAT use cases.

	Study type	Field	Search engine and source scrapers	Number of search results	Evaluation view	Classifier or extension	Reference
Conducted with current RAT version	Classification study (automatic)	Political science	Organic results from Google	1,372	None	SEO classifier	Hinz, Sünkler & Lewandowski (2023)
	Content analysis	Library and information science	Results from SUB University Hamburg library system	5,948	Biases in library catalogues	None	unpublished student work^*^
		Media and communication science	Organic results from Google	5,710	None	Query sampler extension	Ekström & Tattersall Wallin (2023), Haider et al. (2023)
	Source distribution analysis	Information science	Organic results from Google, Bing, DuckDuckGo, and MetaGer	141,480	None	None	Yagci et al. (2022)
			Organic results from Google	378,581	None	None	Norocel & Lewandowski (2023)
Conducted with previous RAT version	Interactive Information retrieval study	Information retrieval	Organic results from Google and Bing	2,288	Relevance assessments	Search logger extension	Sünkler & Lewandowski (2017)
	Retrieval effectiveness study	Information retrieval	Organic results from Google and Bing	22,000	Relevance assessments	None	Lewandowski (2015)
			Organic results form Million Short	750	Relevance assessments	None	Schaer et al. (2016)
			Organic results from Google and Bing	20,000	Relevance assessments	None	Lewandowski (2013)
			Results from EconBiz library system	35,158	Relevance assessments	None	Behnert (2015), Behnert & Plassmeier (2016)
Not conducted with RAT, but could have been supported by RAT	Information quality study	Health	Organic results from Google, Yahoo, and Bing	49	Quality assessments	None	Janssen et al. (2019)
		Information science	Organic results from Google	60	Quality assessments	SEO classifier	Schultheiß (2023)
	Classification study (manually)	Health	Organic results from Google	540	Classification of source types	None	Döring (2017)
		Media and communication science	Organic results from several search engines	3,350	Classification of fake news	None	Mazzeo, Rapisarda & Giuffrida (2021)
	Content analysis	Health	Organic results from Google	227	*E.g.*, portrayal of immune boosting	None	Rachul et al. (2020)

**Notes.**

* This study serves as a representative of the large number of student work supported by RAT. For more student work, see https://searchstudies.org/research/rat/.

### Retrieval effectiveness studies

As described in the Introduction, the early version of RAT was developed for conducting retrieval effectiveness studies and was consequently referred to as the *Relevance* Assessment Tool. One exemplary study conducted with this early RAT version is the work of Lewandowski (2015) on the retrieval effectiveness of Google and Bing. Using a crowdsourcing approach, study participants evaluated results for 1,000 queries with informational and navigational intent each. The query samples were taken from a transaction log of the T-Online search engine. The results showed that Google outperformed Bing for both search intents tested, although results for navigational and informational queries differed significantly. The main RAT components of RAT Frontend (Researcher View for designing the study, Evaluation View for completing the assessments) and RAT Backend (Search Engine Scraper and Source Scraper) are used in retrieval effectiveness studies.

### Interactive information retrieval studies

Studies from the field of interactive information retrieval (IIR) require the logging of user interactions with search engines. An extension called Search Logger (Singer et al., 2011) was used for that purpose. The work by Sünkler & Lewandowski (2017) serves as an example of an IIR study conducted with the early RAT version. In the study, *N* = 64 subjects worked on search tasks while the Search Logger recorded data such as the search engine used, the search queries entered, and the search results selected. RAT then collected the first ten results for each search query entered by the participants and asked the same participants to evaluate the relevance of the collected results. This approach is particularly interesting as it allows researchers to ask study participants for relevance judgments to results for the same participants’ queries (instead of asking them to assess results for queries predefined by researchers). This allowed analyses such as measuring the precision and relevance of selected search results. The study findings show that subjects primarily selected results they later assessed as relevant, indicating that snippets provided by the search engines prove useful in IIR settings.

### Classification studies

RAT supports both manual (*i.e.,* conducted by study participants or researchers themselves) and automatic search result classifications. The works of Döring (2017), Mazzeo, Rapisarda & Giuffrida (2021), and Ballatore (2015) provide examples of human-conducted classification studies that could have benefited from RAT had it been available at the time.

In these studies, researchers faced methodological challenges. Döring (2017) and Ballatore (2015) manually collected, saved, and classified search results—a labor-intensive process prone to inconsistencies. Ballatore (2015) specifically invested substantial effort attempting to minimize temporal and spatial biases by utilizing the Tor browser and conducting multiple searches with no integrated tool support. Similarly, Mazzeo, Rapisarda & Giuffrida (2021) had to develop a custom approach to collect search results for their manual labeling process.

Had RAT been available, these researchers could have streamlined their workflows considerably. Instead of developing ad hoc methods, they could have employed RAT’s integrated process for scraping data from various locations and time periods while applying classification procedures tailored to their specific criteria. The benefits would have been substantial:

RAT improves upon these earlier classification methods in several key ways. Unlike the fragmented approaches these studies had to devise, RAT offers a consistent, replicable framework that lowers methodological inconsistencies throughout the research process. These previous studies depended on separate data collection, storage, and classification solutions, leading to potential data transfer errors and compatibility issues. RAT’s integrated environment eliminates these problems entirely.

Furthermore, while Ballatore (2015) had to manually configure Tor for geographic variation and schedule repeated searches, RAT’s built-in scheduling and proxy setup capabilities would have automated these processes. The platform’s structured assessment interface would have enabled a more consistent application of classification criteria and simpler validation of inter-rater reliability when multiple researchers participated in studies like Döring (2017).

In practice, RAT would have allowed these researchers to categorize search results based on their specific criteria—source types (Döring, 2017), ideological bias (Ballatore, 2015), or misinformation indicators (Mazzeo, Rapisarda & Giuffrida, 2021)—with greater efficiency and reliability. Studies without external participants would have benefited from RAT’s Evaluation View for direct classification. The proxy settings and scheduling features would have provided systematic data collection capabilities far beyond what was possible with their manual methods.

For a concrete example of RAT’s capabilities in automatic classification, we can refer to the study by Hinz, Sünkler & Lewandowski (2023), which actually did use RAT. In this study, the authors analyzed SEO usage on candidates’ personal websites during the 2021 German federal election. The SEO classifier, described in the Classifier section, was used to identify SEO on 93% of the 1,372 websites examined. Unlike the earlier examples, this study did not require evaluation by human assessors, as the SEO classification was performed automatically, forming the final study result.

### Content analyses

RAT can also support content analyses based on search results. RAT can demonstrate its strength in content analyses by automatically collecting the search results, integrating all necessary study steps, and eliminating the need for additional coding tools.

Two studies involving content analyses have already been conducted with RAT. In a student research project (unpublished), biases in library catalogs were explored. For this purpose, 5,948 search results were scraped from SUB University Hamburg library system for 100 thematically diverse queries. The search results were coded by author gender, location of the item, and other categories. A second study conducted with RAT is the work by Haider et al. (2023). For the Swedish term for wind power (*vindkraft*), the *query sampler* extension described by Schultheiß et al. (2023) generated 252 queries for which RAT scraped 5,710 Google results. The authors analyzed the queries for monthly average searches and classified the search results according to source types, such as energy companies or news media. Additionally, the authors coded news articles regarding their favorable, general, and unfavorable depictions of wind power.^1^ 1Note that for the Haider et al. (2023) study, the data initially provided by RAT for query generation and initial scraping was supplemented by a separate scraping process for news articles conducted by the authors. Since RAT could have performed both steps, this distinction is not further elaborated in our overview of studies (*e.g.*, Table 4).

A content analysis that would have benefited from RAT is the work of Rachul et al. (2020) from the health field. The authors manually collected 227 COVID-19-related search results from Google in Canada and the US. The results were coded, among other things, according to how immune-boosting and supplements are portrayed and whether an immune-boosting product or service is being sold or advertised. Utilizing RAT would have benefited the study by automatically gathering the search results, removing the need for data collection by hand. Furthermore, the content analysis could also have been performed within the RAT interface, enhancing the overall efficiency of the analysis process.

### Information quality studies

Studies on the quality assessment of online information are prevalent in the health field. Researchers typically use DISCERN (Charnock et al., 1999) or other standardized questionnaires to evaluate patient information. DISCERN contains 16 questions, such as “Is it balanced and unbiased?” which are to be answered on a scale from 1 (no) *via* 3 (partially) to 5 (yes). DISCERN is intended for experts, *i.e.,* information producers and health professionals, as well as laypersons, *i.e.,* patients (Charnock et al., 1999). DISCERN is widely used, for example, in studies on quality assessment of patient information on the web about prostate cancer (Janssen et al., 2019). Questionnaires such as DISCERN can be easily integrated into RAT as a template that can be selected by the researchers in the study design.

Besides health information, information quality can also be investigated in any other research context, such as in the field of information science or media communication. For example, RAT can assist in assessing whether there are quality differences between web pages that use SEO and those that do not (Schultheiß, 2023). This study example demonstrates the flexibility of RAT, allowing the usage of its components like the SEO classification at different stages in study design. The study by Hinz, Sünkler & Lewandowski (2023) shows that the SEO classification of politicians’ websites was the final study result without a subsequent user evaluation. In contrast, in the case of the study example by Schultheiß (2023), the SEO classification serves as an intermediate step that determines which search results are forwarded for quality evaluation by study participants.

### Source distribution analyses

Another study type supported by RAT is source distribution analyses, such as comparisons between search engines and countries where no result evaluation by human participants is included.

The study by Yagci et al. (2022) analyzed the source diversity of Google and alternative search engines and to what extent the root domains overlap between the top results from these search engines. For 3,537 queries taken from Google Trends in Germany and the US, the top 10 search results from Google, Bing, DuckDuckGo, and MetaGer were scraped, totaling 141,480 results. Analysis of the root domains revealed a significant presence of Wikipedia and news services across the search engines. In addition, only a tiny overlap was found between Google and the alternative search engines (24 to 28%). A higher overlap was found among the alternative search engines (up to 70%), which might be explained by their integration of results from Bing. The study by Norocel & Lewandowski (2023) analyzed search queries on a continuum between mainstream and extreme-right vocabularies for Germany and Sweden. For 21,105 queries, 378,581 Google results were scraped using RAT. An evaluation of the source types showed that popular sites for user-generated content, such as Facebook, are highly ranked for all queries in both countries, allowing extreme-right fringe entities to become visible with their content in the search results.

Currently, RAT specifically assists source distribution analyses by automatically measuring domain overlap between search engines as part of the analysis module. The results of the overlap analysis are shown in Researcher View (see ‘Researcher View’).

## Discussion and conclusion

In summary, RAT enables the efficient conducting of diverse studies based on results from search engines and other search systems. The software has the potential to transform the way researchers work with search data, ranging from retrieval effectiveness studies in information retrieval to information quality assessments in domains like health information. By automating processes traditionally performed manually—such as collecting and distributing results to study participants—RAT creates significant efficiency gains for researchers.

While RAT significantly enhances the research scale through automation, we recognize that larger datasets do not automatically yield better research. RAT’s methodological advantages stem from systematically addressing common biases in human-conducted collection. The system’s error recovery mechanisms, standardized assessment interfaces, and comprehensive documentation ensure data quality beyond quantity. The ability to capture search results at a specific moment across all queries eliminates the temporal inconsistency that plagues traditional collection approaches spread across days or weeks. This temporal consistency is particularly valuable in studying rapidly evolving search landscapes where results can change significantly over short periods.

RAT’s primary strength lies in enabling research questions that were previously impractical to investigate. Studies examining source distribution patterns across multiple search engines or tracking result consistency over time require analyzing tens or hundreds of thousands of results. This scale would be prohibitively resource-intensive with non-automated methods. RAT’s approach combines the methodological advantages of automated collection with the capabilities of human assessment, allowing researchers to apply their expertise to analyzing patterns rather than spending it on repetitive collection tasks.

Traditional methods may still be preferable for small-scale exploratory research, specialized assessments requiring deep domain expertise, or studies focusing on unique interface elements that automated tools might miss. RAT is designed to complement rather than replace researcher judgment, providing a methodological infrastructure that supports both quantitative scale and qualitative depth while reducing known sources of bias.

A critical consideration in automated data collection is whether the process introduces methodological biases. For instance, automated scraping could favor specific query structures or miss context-dependent results that human researchers might notice. RAT addresses these concerns through several mechanisms. First, the system allows researchers to define queries precisely as they would be entered by human users, preserving natural language patterns and query structures. Second, RAT’s proxy configuration enables results collection from different geographic locations, preventing the regional bias that often affects single-point collection. Third, the software captures screenshots and full HTML content, preserving contextual elements like featured snippets, knowledge panels, and multimedia content that might be missed by API-based collection. Finally, RAT’s documentation of the entire collection process creates transparency that allows researchers to identify and account for any systematic limitations in their methodology. These safeguards ensure that automation enhances rather than compromises data quality, while the standardized collection process reduces the subjective biases affecting manual collection methods.

RAT supports both human-based and automatic analysis pathways. As a web-based platform, human participants can be easily invited to participate in studies. The Assessment Interface offers researchers a structured environment to evaluate results obtained from search engines, either through participant recruitment or direct researcher assessment. The software provides integrated modules like the SEO classifier and overlap analysis for automatic analysis. The modular architecture facilitates straightforward integration of new analysis capabilities, demonstrating the framework’s adaptability. Researchers can also extend RAT’s functionality by adding custom scrapers for search systems not yet supported in the core software.

A key strength of RAT is its accessibility to researchers beyond technical disciplines. While the system offers sophisticated capabilities for information science researchers, we designed the platform to support social scientists and researchers from other fields without requiring advanced technical knowledge. Extensive usability testing and active support for researchers from diverse disciplines help promote search engine data analysis as a viable research approach across fields. Offering RAT as a service is particularly important, as many researchers may be unwilling or unable to manage their installation, especially when conducting a single study. These accessibility measures strengthen the interdisciplinary community of search engine researchers.

To ensure that RAT remains viable over the long term, we invest in building both a user community and a developer ecosystem. By encouraging extensions to RAT and inviting participation in software maintenance, we create a sustainable model for the platform’s evolution. Every component of RAT is open source, enabling any interested researcher or developer to contribute to or build upon the framework.

While highlighting RAT’s capabilities, we must acknowledge the limitations of our approach. Scraping search engine results requires ongoing maintenance as search providers frequently modify the structure of their search engine result pages, necessitating continuous scraper adjustments. Capturing full content from result documents presents additional challenges, particularly when websites implement dynamic content loading through user interactions like scrolling. This is especially prevalent in news services and social media platforms that progressively load content as users navigate pages.

In future development, we plan to integrate RAT with complementary research tools to create a more comprehensive ecosystem. Integration with participant recruitment platforms like Prolific would streamline participant management for researchers conducting evaluation studies. Connections to data analysis platforms such as KNIME would enhance post-collection analysis capabilities. As search engines increasingly incorporate large language model-generated responses alongside conventional results, we aim to develop specialized scrapers for collecting and analyzing these new result formats.

These ongoing developments reflect our commitment to evolving RAT alongside changing search technologies and research methodologies, ensuring that the platform remains valuable to researchers investigating how search engines shape information access in the digital age.

## Additional Information and Declarations

### Data Availability

The following information was supplied regarding data availability: The source code is available at Zenodo: Lewandowski, D., Sünkler, S., & Yagci, N. (2024). Result Assessment Tool (RAT) (1.2). Zenodo. https://doi.org/10.5281/zenodo.14049811.
